# Trophic transfer of essential elements in the clownfish *Amphiprion ocellaris* in the context of ocean acidification

**DOI:** 10.1371/journal.pone.0174344

**Published:** 2017-04-11

**Authors:** Hugo Jacob, Simon Pouil, David Lecchini, François Oberhänsli, Peter Swarzenski, Marc Metian

**Affiliations:** 1 International Atomic Energy Agency, Environment Laboratories, 4a, Quai Antoine 1er, Principality of Monaco, Monaco; 2 USR 3278 CNRS-EPHE-UPVD, Paris Sciences Lettres (PSL), Université de Perpignan via Domitia, Perpignan, France; 3 Littoral Environnement et Sociétés (LIENSs), UMR 7266, CNRS-Université de La Rochelle, 2 rue Olympe de Gouges, La Rochelle, France; 4 Laboratoire d'Excellence "CORAIL", Moorea, French Polynesia; Universite de La Rochelle, FRANCE

## Abstract

Little information exists on the effects of ocean acidification (OA) on the digestive and post-digestive processes in marine fish. Here, we investigated OA impacts (Δ pH = 0.5) on the trophic transfer of select trace elements in the clownfish *Amphiprion ocellaris* using radiotracer techniques. Assimilation efficiencies of three essential elements (Co, Mn and Zn) as well as their other short-term and long-term kinetic parameters in juvenile clownfish were not affected by this experimental pH change. In complement, their stomach pH during digestion were not affected by the variation in seawater pH. Such observations suggest that OA impacts do not affect element assimilation in these fish. This apparent *p*CO_2_ tolerance may imply that clownfish have the ability to self-regulate pH shifts in their digestive tract, or that they can metabolically accommodate such shifts. Such results are important to accurately assess future OA impacts on diverse marine biota, as such impacts are highly species specific, complex, and may be modulated by species-specific metabolic processes.

## Introduction

The absorption of increased atmospheric CO_2_ concentrations by the oceans is changing seawater chemistry, with forecasts estimating a drop of 0.3–0.4 units in ocean pH by the year 2100 [1]. This process is known as ocean acidification (OA) and has been shown to affect vital functions of many marine biota [2]. While the majority of research regarding the effects of CO_2_-driven ocean acidification have focused on calcifying marine organisms, fewer studies exist on the impacts of OA on fish health [3]. Effects of increased *p*CO_2_ on fish vary from species to species, life stage, and biological processes [4]. For example, Moran and Stottrup [5] found weight and growth rates substantially reduced in juvenile Atlantic cod (*Gadus morhua*) exposed to increasing levels of *p*CO_2_. Conversely, other studies have showed that elevated levels of *p*CO_2_ had no clear effect on growth rates in juvenile spiny damselfish, *Acanthochromis polyacanthus* [6] and juvenile scups, *Stenotomus chrysops* [7]. Furthermore, Welch and Munday [8] found that high level of *p*CO_2_ increased the reproductive output in the clownfish *A*. *percula*, but decreased the reproductive output in *A*. *polyacanthus* in similar conditions.

Among the biological processes studied in the context of OA research on marine fishes, digestion has received little attention to date. Frommel et al. [9,10] found morphological and physiological impairments in the digestive system of fish during early life stages under ocean acidification scenarios, but no direct correlations were made to physiological digestion mechanisms. However, a few recent studies that examined the digestive process found strong evidence that ocean acidification may affect the digestive capabilities of some fishes. For example, Rosa et al. [11] showed that hypercapnic conditions led to a substantial decrease in digestive enzyme activity; 42% in the activity of trypsin and 50% in the activity of alkaline phosphatase in the juvenile of the Bamboo shark *Chiloscyllium punctatum*. Moreover, Pimentel et al. [12] showed that similar conditions led to a decrease in both pancreatic (up to 26.1% for trypsin and 74.5% for amylase) and intestinal enzymes (up to 36.1% for alkaline phosphatase) of the post-metamorphic larvae of the flatfish *Solea senegalensis*. However, if new observations indicate that ocean acidification may interfere with fish digestion, the association between a decrease in enzyme activity and an essential element’s assimilation deficiency has not yet been investigated. One critical parameter for understanding metal trophic transfer in fish is the assimilation efficiency (AE) of an element from ingested food. If derived under controlled experimental conditions, AE is a first-order physiological parameter that can be compared quantitatively among different trace elements, organisms, food types, or environmental conditions [13].

The present study aimed to investigate the possible effects of ocean acidification on the assimilation efficiency of three essential elements using radiotracers (^57^Co, ^54^Mn, ^65^Zn) in juvenile clownfish *Amphiprion ocellaris*. Juveniles were exposed to projected future *p*CO_2_ levels over the next two centuries (pH 7.5) [1] as well as present-day conditions (pH 8.0) and their trophic transfer and assimilation efficiency (AE) for each element was compared between the two conditions using radiotracer techniques. It is worth noting that the selected low pH condition may also represent a low pH that can occasionally occur at the current time in coral reef lagoon [14].

## Material and methods

### Origin and acclimation of the organisms

In February 2016, one hundred juvenile clownfish *A*. *ocellaris* just under two months old were obtained from a fish farm (Ecorecif, France) and transported to the IAEA Radioecology Laboratory in Monaco. Juveniles were randomly separated into two groups and acclimatized for seven weeks using two 20-L aquaria, configured as follows: (open circuit, water filtration = 0.45 µm; salinity = 38; temperature = 25.8 ± 0.3°C; renewal of seawater = 70% h^-1^; photoperiod = 12h / 12h), at either pH 8.0 ± 0.07 (*p*CO_2_: 456 μatm) and 7.5 ± 0.06 (*p*CO_2_: 1775 μatm). The pH_NBS_ was monitored every 15 minutes in each aquarium to within ± 0.05 pH_NBS_ units using a continuous pH-stat system (IKS, Karlsbad) that purged a pre-set rate of pure CO_2_ into the aquaria. Temperature in each aquarium was also monitored with the same system. The pH probes were regularly calibrated using Tris-HCl and NBS buffer solutions [15]. Total alkalinity was measured by titration using Methrom 809 Titrando® calibrated with NBS buffers, Tris-HCl (Dickson, Batch #137) and standards (Dickson, Batch #150). The *p*CO_2_ was determined from pH, temperature and total alkalinity measurements using the R package seacarb [16].

### Kinetics of stomach pH in juvenile clownfish after a single feeding

The influence of seawater pH on juvenile clownfish was investigated since stomach pH can directly affect the digestive process [17] and it may thus affect assimilation efficiencies of essential elements by fish. For this purpose, a total of 24 acclimatized, juvenile clownfish were selected for each pH treatment (control condition: 0.51 ± 0.18 g; acidified conditions: 0.57 ± 0.17 g) and were acclimated to a single 9:00am feeding (pellets) for two weeks prior to the experiment. The experiment consisted of feeding fish *ad libitum* to characterize the acidification capacity in the fish stomach in two different conditions. Three fish (from both conditions) were subsequently sampled at various times over 8 hours. Three other individuals were sampled right before the feeding in order to determine the preprandial level of stomach pH. For pH determinations, measurements were conducted in living animals using a pH microelectrode (ThermoScientific, 9810BN). In order to minimize adverse impacts on the welfare of fish during this experiment, fish were anaesthetized using Eugenol and, immediately after anesthesia, the tip of the microelectrode was inserted into a small slit made in the stomach [17]. The CRIOBE committee approved the sampling protocol and gave the ethical permission CRIOBE-Fish-2016-012. At the end of the experiment, the fish were sacrified by decapitation and disposed of following IAEA radioactive waste procedures. A statistical comparison between pH values as a function of time for the two conditions was made using the two-tailed Mann-Whitney U test.

### Long-term depuration of ^57^Co, ^54^Mn, and ^65^Zn in juvenile clownfish

A total of 15 acclimatized juvenile clownfish were selected for each pH treatment (control condition: 0.32 ± 0.05 g; acidified conditions: 0.34 ± 0.06 g). Juveniles were transferred into two preconditioned 20-L aquaria that were set up in conditions similar to those of acclimatization tanks (with pH set to 7.5 ± 0.05 and 8.0 ± 0.08, temperature = 25.5 ± 0.3°C; and the same seawater renewal rate). During the two weeks prior to exposure to radiolabeled food, the fish were fed daily with pellets of the same size as those used in the experiment.

The experiment consisted in one single exposure of fish to radiolabeled feed (single-feeding method; e.g., [18]). Clownfish were fed *ad libitum* and all uneaten pellets were removed after 5 min. From that moment, all fish (including control fish; fed with non-radiolabelled food in order to assess potential seawater contamination) were radioanalysed regularly to monitor the depuration kinetics of radiotracers over a period of 20 days. During the entire experiment, there were no negative effects observed on the well-being of the specimens.

### Short-term depuration of ^57^Co, ^54^Mn and ^65^Zn in juvenile clownfish

In order to get a best description of the early process of trophic transfer of the ^57^Co, ^54^Mn and ^65^Zn in juvenile clownfish, a short-term experiment (<1d) was conducted. A total of six acclimatized clownfish were selected for each treatment (control condition = 0.38 ± 0.1 g; acidified conditions: 0.40 ± 0.1 g), and transferred to two 20-L aquaria in conditions identical to those of the acclimatization tanks. For this experiment (method being similar to long-term exposure one), individuals were radioanalysed at various times during one day (3, 6, 9, 12 and 24h).

### Radiotracers and counting

Uptake and depuration kinetics of the three essential elements (Co, Mn, and Zn) were determined using matched radiotracers (^57^Co as CoCl_2_ in 0.1 M HCl, [*t*_*1/2*_ = 271.8 days]; ^54^Mn as MnCl_2_ in 0.5 M HCl, [*t*_*1/2*_ = 312.2 days]; ^65^Zn as ZnCl_2_ in 0.1M HCl, [*t*_*1/2*_ = 243.9 days]) purchased from Isotope Product Lab., USA. The radioactivity of the tracers was quantified using high-resolution γ-spectrometer systems that consisted of a suite of four high purity germanium (HPGe) N or P type—detectors (EGNC 33-195-R, Canberra® and Eurysis®) controlled by a multi-channel analyser and a computer equipped with spectral analysis software (Interwinner® 6, Intertechnique).

The radioactivity of each isotope was determined using calibrated standards of the same geometry. Measurements were corrected for background activity and radioactive decay. Live clownfish specimens were placed into custom designed holding tubes filled with non-contaminated seawater for gamma spectrometric analyses. This method maintained sufficient dissolved oxygen levels while minimizing counting errors due to geometry effects from movement of the live specimen during counting [19]. Counting time was adjusted to obtain propagated counting errors typically less than 5%; run times were typically 10–20 min for whole specimen radioanalysis (longer count times (up to 40 min) were required towards the end of long-term depuration experiment), which was also shown to not affect the specimen’s wellbeing. Tests were performed prior to the experiments by placing fish into identical counting conditions to assess their behavior during counting (e.g. water temperature was stable and dissolved O_2_ concentrations were always > 3 mg L^-1^).

### Radiolabelled processed feed pellet

Radiolabeling of the compound food pellets was performed as described by Pouil et al. [20] using the selected radiotracers described above. Briefly, 1.2 gr of pellets were dipped for 1 h in 1 mL of seawater spiked with ^57^Co, ^54^Mn and ^65^Zn. The food pellets were then dried for 48 hr (50°C) to prevent nutritional loss and mold growth. Potential discharge of the radioisotopes into seawater, which may then lead to a double exposure of the fish (food and water) was tested and confirmed not to be an issue as long as the fish consumed the food pellets within 1 min. Although these tests confirm the single-pathway contamination (*viz*. food) of the fish, additional clownfish were used as controls against possible contamination by seawater.

### Kinetic data from the single-feeding experiment

Observation of depuration kinetics of these radiotracers were fitted using nonlinear regression routines and iterative adjustment using Statistica software 7.0. These kinetics were best fitted using a one- or two-component exponential model [21] or a two-component model that includes an exponential component and a constant as described by Pouil et al. [20,22]. A comparison between the remaining activities at different times for the short- and long-term experiments was made using the two-tailed Wilcoxon-Mann-Whitney test. The level of significance for statistical analyses was always set at α = 0.05. All the statistical analyses were performed using R software (R-3.2.1).

## Results and discussion

In order to evaluate the influence of pH on the absorption kinetics of essential elements in *A*. *ocellaris*, depuration of ^57^Co, ^54^Mn and ^65^Zn were followed after a single feeding using radiolabeled pellets for both 24 hours and 20 days. The average activities in pellets were 10.5 ± 0.5 kBq g^-1^ of ^57^Co, 5.4 ± 0.3 kBq^.^g^-1^ of ^54^Mn, and 5.2 ± 0.3 kBq g^-1^ of ^65^Zn.

**Fig 1:**
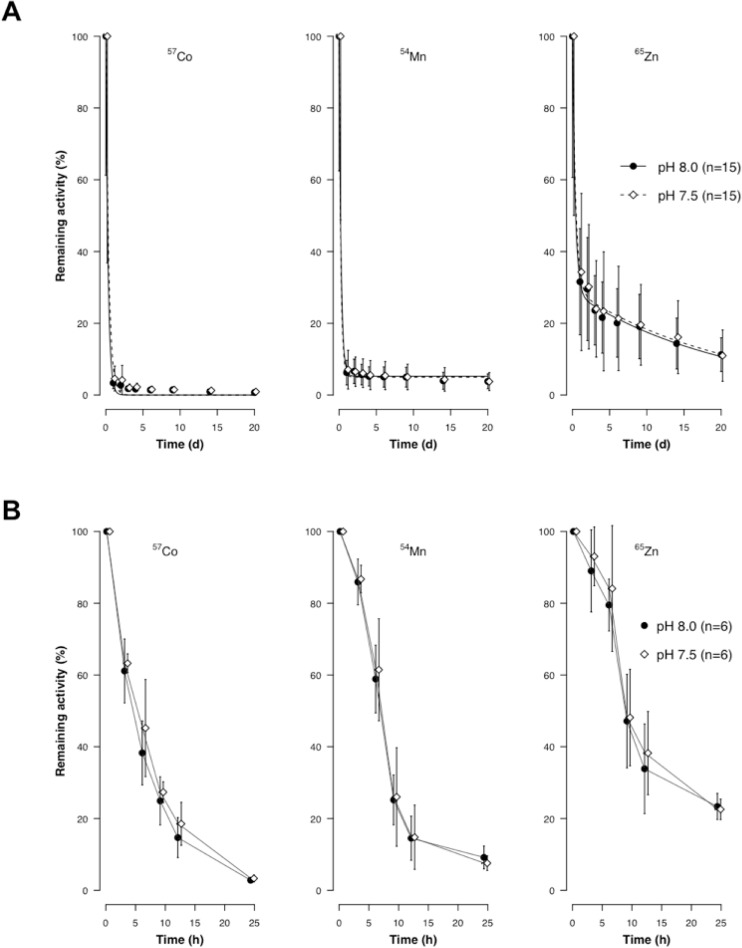
**Short-term depuration experiment: Influence of pH on the depuration of Co, Mn and Zn during 20 days (A) and 24 hours (B) in juvenile clownfish *A*. *ocellaris* fed with radiolabelled pellets (single feeding approach).** Values are means ± SD (See details in S1 File).

During the 20-day experiment, depuration kinetics of ^57^Co, ^54^Mn, ^65^Zn in control and acidified conditions were described by a single exponential component model for ^57^Co, a double exponential component model for ^65^Zn, and a dual component model with a constant for ^54^Mn (Table 1 and Fig 1A). A large proportion (70–99%) of ingested radiotracer has been associated with the short-term depuration. This component was characterized by a rapid loss (*t*_*b1/2s*_ < 1) independently of pH conditions. At the end of the depuration period (20 days), the total retained activity (*i*.*e*. assimilation efficiency: AE) was respectively 5 ± 1% and 5 ± 5% for ^54^Mn, and 28 ± 4% and 29 ± 5% for ^65^Zn in control and acidified treatments, however ^57^Co was not assimilated in both conditions (Table 1). There was no significant difference in the percentage of activity remaining between the two pH conditions for each element (Mann-Whitney U test, *U* = 91–146, *N1* = 15–16, *N2* = 16, *P* > 0.1). For the short-term depuration experiment (Fig 1B), there were also no significant differences in the remaining activities of individuals followed at each radioanalysed times (3, 6, 9, 12 and 24h) between pH conditions (Mann-Whitney U test, *U* = 12–26, *N1* = 6, *N2* = 6, *P* > 0.1).

**Table 1:** Long-term depuration experiment: estimated depuration kinetic parameters of ^57^Co ^54^Mn and ^65^Zn in juvenile *A*. *ocellaris* exposed to radiotracers by radiolabelled pellets (single feeding approach) in two pH treatments (7.5 and 8.0) and held for 20 days in unspiked water.

Isotope	pH	Model type	First component (Short lived)			Second component (Long lived)			
			k_es_	A_0s_ ± ASE	T_b1/2s_ ± ASE	k_el_ ± ASE	AE± ASE	T_b1/2l_	R^2^
^57^Co	8.0	S	3.32 ± 0.92***	99.99 ± 3.09***	0.21 ± 0.06	-	-	-	0.87
	7.5	S	2.50±0.59***	99.95±4.55***	0.28±0.07	-	-	-	0.74
^54^Mn	8.0	DC	4.33±2.75*	95.02±3.23***	0.16±0.10	-	4.98 ± 1.15***	∞	0.87
	7.5	DC	3.91±2.53*	94.77±4.71***	0.18±0.11	-	5.22 ± 4.72***	∞	0.76
^65^Zn	8.0	D	2.71±1.19*	71.34±5.46***	0.26±0.11	0.05 ± 0.02**	28.65 ± 3.78***	14.15	0.74
	7.5	D	2.34±1.10*	70.61±7.53***	0.30±0.14	0.05± 0.02*	29.36 ± 5.22***	14.75	0.59

S: depuration model with one exponential component (A_t_ = A_0_. e^-ke.t^); D: depuration model with two exponential components (A_t_ = A_0s_. e^-kes.t^ + A_0l_. e^-kel^); DC: Two-component depuration model with constant (A_t_ = A_0_. e^-kes.t^ + A_0l_ where A_0l_ = AE); k_es_ and k_el_: depuration rate constant (d^-1^) according to the short- and the long-lived exponential component (d^-1^); A_0s_ and A_0l_ (= AE): remaining activity (%) according to the short- and the long-lived exponential component; T_b1/2_: biological half-life (days); ASE: asymptotic standard error; R^2^: determination coefficient.

*** p < 0.001

** p < 0.01

* p < 0.05

**Fig 2:**
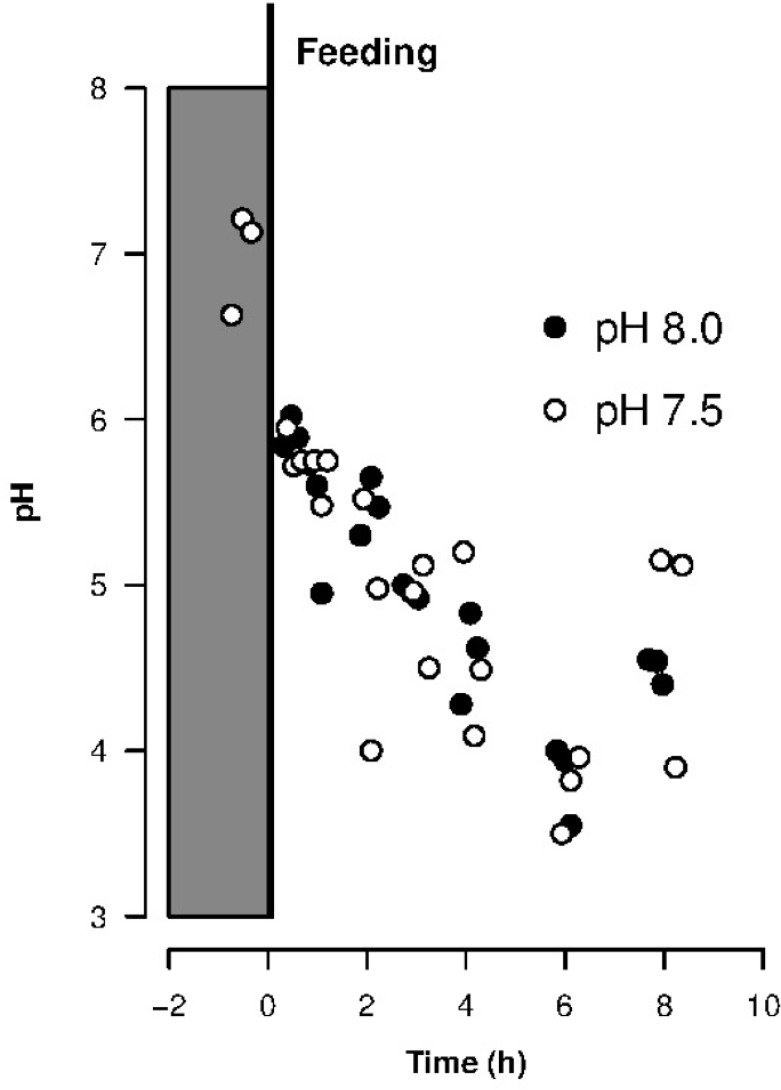
Influence of environmental pH condition on stomach pH of juvenile clownfish *A*. *ocellaris* before a single feeding and during 8 hours after feeding. Values are individual pH measurements (See details in S1 File).

Being one of the first studies to investigate the effects of increased *p*CO_2_ on the trophic transfer of essential elements in fish, it is premature to broadly generalize the absence of direct effects found here to other fishes. Indeed, sensitivities may vary among species, especially among species with between different life cycles; coral reef species appears to be particularly tolerant to the effects of ocean acidification. Many studies have found no effect of increased *p*CO_2_ on growth rates in juvenile coral fish species [6,23], which further corroborate the absence of effect on assimilation in our study (stomach’s pH of juvenile *A*. *ocellaris* was not affected, Mann-Whitney U test, *U* = 3–12, *N1* = 3, *N2* = 3, *P* > 0.1; Fig 2). This resistance may result from physiological adaptations from residing in a variable pH environment, such as coral reefs [24]. In our study, enzyme activity was not measured in the digestive tract while stomach pH was: this parameter regulates the activity of various enzymes such as pepsin [25] and it was not modified by an increase of *p*CO_2_ in seawater (Fig 2).

In past studies where the effects of ocean acidification on digestive enzyme activity was studied in the flatfish *Solea senegalensis* [12] and the Bamboo shark *Chiloscyllium punctatum* [11], the fitness and the survival of the species were also negatively impacted by ocean acidification [26, 27]. One possible explanation for the differences in response to ocean acidification observed between these two groups of species (coral fish *vs*. flatfish and benthic sharks) might come from their distinct metabolic rates. Indeed, Melzner et al. [28] suggested that marine animals with higher metabolic rates might be less affected by ocean acidification due higher extracellular *p*CO_2_ values and advanced metabolism for the elimination of CO_2_ and associated acid-base disturbances. Clownfish have higher metabolic rates many other fish species, including flatfish and bamboo shark [29], which should make them more tolerant to hypercapnia. Future studies should therefor focus on the effects of increased *p*CO_2_ on food assimilation for lower metabolic rate fish species.

While species with higher metabolic rate may better cope with changes of *p*CO_2_ in their environment, it is well reported that a series of fish physiological processes can be disturbed by higher *p*CO_2_ [4]. Among the affected processes, it is interesting to focus on the acid-base balance and the ionoregulation in the context of the trophic transfer of essential elements. Indeed, dietary trace element absorption occurs mainly in the intestine [30,31], and in marine fish (drinking water species), the intestine is also involved in acid-base balance and the ionoregulation (with gills and kidney [32,33]). Hereto, even if previous studies provided evidence of disturbances caused by *p*CO_2_ on these processes in the fish intestine [34,35], our results indicated that it does not affect dietary essential element transfer in clownfish.

The current study presents intriguing findings in the response of one fish species to the effects of OA through the trophic transfer of select essential elements. Care needs to be taken in how to interpret and expand on these results. For example, compound feed was used for the juvenile clownfish. Therefore, assimilation efficiencies observed in this study might not reflect actual assimilation of these elements in natural conditions, as artificial food has been found to influence the assimilation of different metals in comparison to natural preys although we used the same type of food for both treatments. Pouil et al. [22] showed that the assimilation efficiencies of essential elements were influenced by the nature of food provided to the flatfish *Scophthalmus maximus*, as the specific physicochemical forms and metabolites will likely play a role in the bioavailability of these elements for predators [36]. Therefore, depending on the experimental design, it is foreseeable that the AE of the essential elements in clownfish might vary depending on the initial treatments. Furthermore, and in connection with ecological relevance of this work, changes in seawater chemistry due to ocean acidification will affect the solubility, speciation, and bioavailability of trace metals in water, biota, and sediment [37]. Therefore, also affecting the bioavailability, accumulation and storage of essential elements; the trophic transfer of elements might thus also change along the food chain. The integration of natural prey, also exposed to increased *p*CO_2_, will provide a more realistic view of trophic transfer mechanisms under such environmental stressors.

## Conclusion

This study revealed no statistically significant differences in the assimilation efficiency of juvenile clownfish exposed to high-level treatments of *p*CO_2_ when compared to the fish in the control treatment. It was also shown that during the rapid depuration phase, no difference was observed between fish placed in the two treatments. Thus, these results suggest that the effects of ocean acidification effects do not appear to affect the trophic transfer of essential elements in juvenile clownfish *A*. *ocellaris*, although recent studies found that hypercapnia may decrease the activity of certain digestive enzymes in juvenile fish, sometimes by as much as 50% [10,11]. If ocean acidification can reduce the enzyme activity of juvenile fish, it may thus not ultimately result in a decrease in assimilation (integrative process). Indeed, some metabolic functions can be affected by a variation of seawater pH (or *p*CO_2_) but, according to the present study, the ultimate process (viz. trophic transfer of essential element) may not.

## Supporting information

S1 ARRIVE Checklist ARRIVE guideline checklist. (PDF)

S1 File Kinetic data for stomach pH and radiotracers remaining activities. Data used for (1) kinetics of stomach pH in juvenile clownfish after a single feeding, (2) long-term depuration of ^57^Co, ^54^Mn, and ^65^Zn in juvenile clownfish and (3) short-term (hourly) depuration of ^57^Co, ^54^Mn and ^65^Zn in juvenile clownfish.
(XLSX)
